# Deconstructing Fitbit to Specify the Effective Features in Promoting Physical Activity Among Inactive Adults: Pilot Randomized Controlled Trial

**DOI:** 10.2196/51216

**Published:** 2024-07-12

**Authors:** Keisuke Takano, Takeyuki Oba, Kentaro Katahira, Kenta Kimura

**Affiliations:** 1 Human Informatics and Interaction Research Institute National Institute of Advanced Industrial Science and Technology (AIST) Ibaraki Japan

**Keywords:** wearable activity tracker, mHealth, mobile health, motivation, physical activity, lifestyle, smartwatch, wearables, Fitbit, exercise, fitness, BCT, behavior change technique, behavior change, motivation, adherence, engagement

## Abstract

**Background:**

Wearable activity trackers have become key players in mobile health practice as they offer various behavior change techniques (BCTs) to help improve physical activity (PA). Typically, multiple BCTs are implemented simultaneously in a device, making it difficult to identify which BCTs specifically improve PA.

**Objective:**

We investigated the effects of BCTs implemented on a smartwatch, the Fitbit, to determine how each technique promoted PA.

**Methods:**

This study was a single-blind, pilot randomized controlled trial, in which 70 adults (n=44, 63% women; mean age 40.5, SD 12.56 years; closed user group) were allocated to 1 of 3 BCT conditions: self-monitoring (feedback on participants’ own steps), goal setting (providing daily step goals), and social comparison (displaying daily steps achieved by peers). Each intervention lasted for 4 weeks (fully automated), during which participants wore a Fitbit and responded to day-to-day questionnaires regarding motivation. At pre- and postintervention time points (in-person sessions), levels and readiness for PA as well as different aspects of motivation were assessed.

**Results:**

Participants showed excellent adherence (mean valid-wear time of Fitbit=26.43/28 days, 94%), and no dropout was recorded. No significant changes were found in self-reported total PA (*dz*<0.28, *P*=.40 for the self-monitoring group, *P*=.58 for the goal setting group, and *P*=.19 for the social comparison group). Fitbit-assessed step count during the intervention period was slightly higher in the goal setting and social comparison groups than in the self-monitoring group, although the effects did not reach statistical significance (*P*=.052 and *P*=.06). However, more than half (27/46, 59%) of the participants in the precontemplation stage reported progress to a higher stage across the 3 conditions. Additionally, significant increases were detected for several aspects of motivation (ie, integrated and external regulation), and significant group differences were identified for the day-to-day changes in external regulation; that is, the self-monitoring group showed a significantly larger increase in the sense of pressure and tension (as part of external regulation) than the goal setting group (*P*=.04).

**Conclusions:**

Fitbit-implemented BCTs promote readiness and motivation for PA, although their effects on PA levels are marginal. The BCT-specific effects were unclear, but preliminary evidence showed that self-monitoring alone may be perceived demanding. Combining self-monitoring with another BCT (or goal setting, at least) may be important for enhancing continuous engagement in PA.

**Trial Registration:**

Open Science Framework; https://osf.io/87qnb/?view_only=f7b72d48bb5044eca4b8ce729f6b403b

## Introduction

### Background

Promoting physical activity (PA) is an urgent mission in modern society, as physical inactivity is a known risk factor for a wide range of illnesses such as diabetes, cardiovascular diseases, and depression [1,2]. Behavior change techniques (BCTs) and lifestyle interventions have been studied extensively, and systematic reviews and meta-analyses have found that, overall, behavioral interventions are effective in promoting PA [3-5]. Notably, many behavioral interventions have been delivered digitally through web applications or smartphone apps (or augmented by online support tools) for recording, giving feedback on, or coaching to promote PA. Thus, eHealth and mobile health (mHealth) interventions are expected to play an important role in promoting PA, especially during and after the COVID-19 pandemic [6,7].

Key players in digital health care research and business are wearable activity trackers, which are (consumer) devices that monitor PA and provide feedback to the wearer. These devices include pedometers, fitness trackers, smartwatches, and smart rings, which are often linked to web applications or smartphone apps that offer individualized dashboards, health messages, social support, and tips to continue exercising regularly (eg, [8,9]). Meta-analyses [10-12] have suggested that activity trackers significantly improve PA with standardized mean difference (SMD)=0.3-0.6, equating to approximately 1800-1850 extra steps per day.

Wearable activity trackers have been found to be well accepted by various populations, including adolescents [13], older adults with chronic diseases [14,15], and cancer survivors [16]. Users typically report positive experiences with activity trackers, which are perceived to be easy to use and useful to track daily (physical) activity. Thematic analyses highlighted enhanced self-awareness among users, which increases motivation to improve or maintain PA [14,15]. Indeed, a pilot trial showed that activity trackers help improve cardiometabolic risk variables in patients with diabetes and could potentially be a motivation tool to increase PA [17,18], particularly with appropriate features and implementations [19].

Fitbit devices are some of the most widely used wearable activity trackers in health care research. A recent narrative review [20] identified 75 published trials using Fitbit, in which a variety of behavior change strategies (eg, competition or challenges, self-monitoring, social support, and goal setting) were implemented. Through these trials, considerable heterogeneity was found in the use of Fitbit. Typically, these trials were designed to investigate the overall effect of a Fitbit-based intervention versus an intervention without Fitbit (or treatment as usual). If not limited to Fitbit, it is often the case with behavioral interventions in which multiple BCTs (ie, 5-10 different techniques [9]) are implemented simultaneously, which makes it difficult to identify which BCTs are active components to improve PA and motivation for PA. Therefore, we aimed to investigate the effects of different BCTs implemented using the Fitbit Sense smartwatch to determine how each technique would improve PA and motivation for PA through a mobile intervention.

The following three BCTs (indexed by the BCT taxonomy v1 [5]) were targeted in this study: (1) self-monitoring, which encouraged participants to monitor and record their step counts each day (*2. Feedback and monitoring* and *2.3 Self-monitoring of behavior*); (2) goal setting, which provided participants with an explicit daily step count goal and feedback on the goal progress (*1.1 Goal setting* and *2.7 Feedback on outcomes of behavior*); and (3) social comparison, which drew the participants’ attention to their peers’ step counts to allow comparison with their own performance (*6.2 Social comparison*). Note that the latter 2 conditions included self-monitoring by nature; without self-monitoring, feedback on goal progress or social comparisons could not be implemented.

We regarded self-monitoring as the control condition (or treatment as usual) because it is the most prevalent BCT implemented in mHealth interventions, and there is robust evidence that self-monitoring increases PA and improves dietary behavior [21,22]. A systematic review on digital implementations of self-monitoring [23] distinguished active self-monitoring, which requires users to reflect on the current state and physical functioning, from passive self-monitoring, which relies on sensors automatically recording (locomotor) acceleration, heart rate, and other physical or environmental information. In this study, we used active self-monitoring (ie, participants manually entered their step count of the day on an online questionnaire platform). This decision was made because (1) we wanted to be sure that each participant consciously checked their steps of the day and (2) the systematic review [23] identified in the literature no intervention study that included wearables to combine active self-monitoring with passive self-monitoring of additional physical information.

Furthermore, a meta-analysis [24] suggested that interventions combining self-monitoring with at least 1 other self-regulatory technique (eg, goal setting or feedback) are more effective than other interventions. A goal setting BCT is implemented in most commercial PA apps—95% set specific and measurable goals, which allow users to evaluate whether and to what extent they achieved the goal [25]. We followed this basic, core implementation, although other components of goal settings (eg, tailoring goal difficulty, scheduling, and action planning) were not considered in this study.

A systematic review [26] found that most of the social features implemented in mHealth interventions involve the delivery of social support or social comparison, which are often combined with the self-monitoring of behavior. The researchers also meta-analyzed the efficiency of mHealth interventions with social features, showing a moderate-to-large size effect, albeit with large heterogeneity (SMD=0.96; *I*^2^=99.6%). A recent analysis of the use patterns of commercial PA apps in a community sample suggested that people typically use only 2 functions within an app, often a combination of self-monitoring and one other function [27]. Given that users highly appreciate the simplicity of an app [28], it is of theoretical and practical importance to clarify how large the additive values of goal setting and social comparison are compared with self-monitoring alone.

### Objectives

In summary, we aimed to test the effects of the 3 BCTs implemented using the Fitbit smartwatch to resolve an answered question in the literature, that is, how each BCT would contribute to improvement in PA levels and motivation in the form of mHealth intervention. A 2 × 3 factorial design was used, with assessment occasions (pre- vs postintervention time points) as the within-person factor and groups (self-monitoring vs goal setting vs social comparison) as the between-person factor. During the 4-week intervention, participants wore a Fitbit Sense to receive 1 of the 3 interventions (ie, self-monitoring, goal setting, or social comparison) and assess their levels of PA (in the form of step count) for 24 hours. They also responded to daily online questionnaires asking how motivated they were regarding PA. The primary outcome was self-reported PA levels assessed before and after the intervention. The secondary outcomes included (1) the stage of change for PA [29], (2) intrinsic and extrinsic motivations for PA, (3) Fitbit-assessed step count, and (4) day-to-day motivations for PA [30]. We expected that the goal setting and social comparison groups would show larger increases in the levels and motivations for PA compared with the self-monitoring group. We did not have a clear hypothesis for a direct comparison between goal setting and social comparison, which was tested in an exploratory manner.

## Methods

### Participants

Invitations were spread to local inhabitants in and around Ibaraki Prefecture (a middle-north region in Japan; February 2023) who had been registered in a database of potential participants. The study was advertised (online) as one that assessed psychological and behavioral data in daily life. The advertisement explicitly stated that participants would be asked to wear the Fitbit for 1 month, but they were blinded to the intervention conditions. In total, 140 individuals were assessed for eligibility, and 70 (50%) met the following inclusion criteria: (1) being aged 18-64 years; (2) expecting no major life change (eg, in a living place or job) before or during the study; (3) being not pregnant or not lactating; (4) being in good health (ie, having no medical, neurological, psychiatric, or other physical illnesses); (5) having not been diagnosed with dysautonomia, depression, mania, bipolar disorder, or facial paralysis in the past; (6) not taking tranquilizers or antihypertensive drugs regularly; (7) not taking hormone replacement therapy or not taking oral contraceptives; and (8) not exercising regularly. Regular PA levels (criterion 8) were assessed using the Stage of Change (SoC) questionnaire for PA (see the *Measures* section), and those who identified themselves as being at the precontemplation, contemplation, or preparation stages were eligible for participation. We assessed SoC instead of PA levels because we targeted motivation and readiness for PA, not only the actual engagement in PA behavior. Among the 70 excluded participants, 60 (86%) were physically more active than criterion 8 (belonging to the action or higher stages of change), and 10 (14%) could not find time for the briefing session of the study. All participants were assumed to have good internet literacy (necessary support was provided at the briefing session by an experimenter).

The sample size was determined after the recommendations of a pilot randomized controlled trial (eg, [31]). We did not perform a rigorous power analysis because we regarded this study as a pilot trial, and there was no good way to determine an expected effect size that could be used for a priori power analysis, particularly for the comparison of goal setting and social comparison. However, the current sample size (n=23 or 24 per group) allowed for the detection of an effect of Cohen *d*=0.87 with α=.05, and power=0.80. A meta-analysis [26] reported SMD=0.96 as the effect of social features implemented in mobile interventions on PA levels albeit high heterogeneity being documented.

### Interventions

Participants were randomly allocated to 1 of 3 groups and received a smartphone and a Fitbit Sense at the beginning of the study (briefing session). The Fitbit app was installed on the smartphones with varying settings for each intervention condition.

In the self-monitoring group, participants received a smartphone with the Fitbit app, with all notification functions turned off, except for low battery warnings. Participants were allowed to see the home display, showing the step count of the day and other physiological information, including sleep, heart rate, and skin temperature. However, the app indicated no step goals to achieve, social functions were disabled, and no push notifications were sent to the devices during the intervention period. Participants received a chat message on their smartphone each evening (at 9 PM) asking them to read the step count of the day displayed on the Fitbit and manually record it on an online questionnaire platform.

Participants in the goal setting group were explicitly informed of the goal to be achieved each day (ie, 9000 and 8500 steps for men and women, respectively) by an experimenter during the briefing session. These goals were set according to the Japanese national health recommendations [32]. During the briefing session, the experimenter instructed the participants to enter the step goal into their Fitbit app to monitor their progress at any time, and the Fitbit Sense notified each participant of their daily goal achievement using vibration.

The participants in the social comparison group were explicitly instructed to read and record the step counts of their peers on the Fitbit app each evening. On the Fitbit app, 5 peers (called “friends”) had been registered before distribution. Participants were not explicitly told who these friends were, but each friend was displayed on the app with a unique participant ID (eg, AIST_01 and AIST_02). These 5 friends were the researchers and research assistants of this project, who wore the Fitbit Sense throughout the period of data collection. The average step count for the 5 friends was 6405-11,449 steps per day. Each participant was paired with the same 5 friends so that there was no variability in the friends’ activity levels between participants. We asked participants to check their friends’ steps each day but did not explicitly ask participants to outperform their friends.

### Measures

We administered the following three types of assessments: (1) pre- and postintervention assessments in the lab, (2) daily in-intervention assessments at home using self-report scales for motivation, and (3) accelerometry (implemented in Fitbit) for step counts (Table 1).

**Table 1:** Overview of the measures and response items.

Type and timing of assessment and measure		Variable and description
**Self-report (in the lab, at pre- and postintervention time points)**		
	Stage of Change questionnaire (at the eligibility screening)	Stage of change, readiness for PA^a^
	International Physical Activity Questionnaire—Short Form	Levels of total PA
	Self-determined Motivation Scale for Exercise	Different motivations for regular PA (intrinsic motivation, external regulation, introjected regulation, identified regulation, integrated regulation, and amotivation)
**Self-report (at home, each evening)**		
	Modified Situational Motivational Scale	Identified regulation, intrinsic motivation, and external regulation
	Step count of the day (all 3 groups)	Read and record the step count of the day, displayed on Fitbit
	Goal achievement of the day (goal setting group only)	Record whether the goal (9000 or 8500 steps) was achieved
	Step count of a peer (social comparison group only)	Record the step count of the Fitbit friend whose count was the highest among the friend group
**Fitbit (at home, at any time)**		
	Step count	Accelerometer based

^a^PA: physical activity.

#### SoC Questionnaire

Readiness for PA was assessed using the Japanese version [33,34] of the SoC questionnaire [29,35]. Participants were classified into 1 of 5 stages (ie, precontemplation, contemplation, preparation, action, and maintenance), selecting the most applicable statement among the following: “I currently do not exercise and do not intend to start exercising in the future” (pre-contemplation); “I currently do not exercise but I am thinking about starting to exercise in the next six months” (contemplation); “I currently exercise some, but not regularly” (preparation); “I currently exercise regularly, but have only begun doing so within the last six months” (action); and “I currently exercise regularly and have done so for longer than six months” (maintenance). We instructed the participants to consider regular exercise as exercising twice or more per week for 20 minutes or longer.

#### International Physical Activity Questionnaire—Short Form

Total PA was assessed using the International Physical Activity Questionnaire—Short Form (IPAQ-SF) [36,37]. The IPAQ-SF includes three dimensions: (1) walking, (2) moderate-intensity activity, and (3) vigorous-intensity activity. Sedentary time was not included in the analyses. Participants reported the number of days and duration spent on each dimension of PA over an average week. The reported weekly minutes of PA (duration×days for each PA dimension) were transformed into hourly metabolic equivalents per week (METs-h/w). This scaling allowed us to explore how many participants adhered to the Japanese public health guidelines for PA, which is 23 METs-h/w for adults [32].

#### Self-determined Motivation Scale for Exercise

The baseline and postintervention levels of motivation for PA were assessed using the revised version of the Self-determined Motivation Scale for Exercise [38], which was developed based on self-determination theory [39]. This 22-item self-report questionnaire covers the following six types of motivations or regulatory styles concerning PA: (1) intrinsic motivation (eg, “Exercising itself is fun”), (2) external regulation (eg, “I exercise because other people will be pleased with me”), (3) introjected regulation (eg, “I feel guilty if I do not exercise”), (4) identified regulation (eg, “I think it is a good way to improve myself”), (5) integrated regulation (eg, “It is essential to my identity and sense of self”), and (6) amotivation (eg, “I do not know why I exercise”). Each construct is assessed using 3- or 4-item subscales, and each item is rated on a 5-point scale (1=not at all true and 5=very true). Cronbach α was >.73 for each subscale.

### Daily Measures: Motivation

A chat message was sent to the participants’ smartphones in the evening (at 9 PM). The message included a link to an online survey platform (Exkuma [40]) where participants rated their motivation for PA. The link was to expire at 2 AM, but because of a technical problem, responses were erroneously accepted even after this due time. All such delayed responses were excluded from analyses. The evening questionnaire included the following 3 items: identified regulation (“I’m feeling that exercising is important for me”), intrinsic motivation (“I’m feeling that exercising is fun”), and external motivation (“I’m feeling that I have to exercise”). Each item is rated on a 7-point scale (1=not at all and 7=very much). Motivation items were adapted from the Situational Motivational Scale [41] for physical exercise [42]. We adjusted the wording for daily or momentary assessments (ie, asking how one is feeling right now) and selected the items with the highest factor loadings in each dimension to minimize the burden on the participants [30]. In addition, we did not include items on amotivation, as our focus was on a pro-PA psychological state, not on the barriers to PA.

### Procedure

Interested participants completed an online screening survey for eligibility. Those identified as eligible were then invited to an online system to schedule an in-person briefing session, where groups of 1-4 participants were formed arbitrarily. These groups were randomly allocated to 1 of the 3 intervention conditions (1:1:1) following a random sequence generated by a trial analyst using R (version 4; R Foundation for Statistical Computing). Experimenters (who conducted the recruitment and assessments) were not involved in this process. Block randomization (batch per group size) was used to prevent imbalances in group allocation. Single blinding was used; participants were not aware of whether they were in an active or control condition or what interventions other participants received. During the briefing session, the participants received instructions about the study, provided informed consent, and completed baseline questionnaires. At the end of this session, the participants received a smartphone (Android OS) paired with a Fitbit Sense. As the data collected via these devices were tagged with participant IDs (but not with their real names), there was no security concerns about personal information.

During the 4-week intervention period, participants were asked to wear a Fitbit Sense on their nondominant wrist for 24 hours. Participants were instructed not to change the settings of the Fitbit app. Each evening, all participants recorded their daily step count on an online questionnaire platform. Those in the social comparison group were also asked to record the highest step count of the day among their friends on the Fitbit app. There was no in-person component in the interventions although technical support (eg, for app login and online questionnaires) was provided by an experimenter at any point of the interventions.

Immediately after the 4-week interventions, the participants were invited to the lab again, returned their smartphones and Fitbit, and completed the postintervention questionnaires. They also took part in a brief in-person interview asking whether participants experienced any adverse events during the intervention period and how they liked Fitbit and each intervention component (ie, self-monitoring, goal setting, or social comparison). At the end of the debriefing session, the participants received 49,000 JPY (US $350) as compensation. Postintervention checks were conducted on the devices right after the interventions were completed. These checks reassured us that (1) the step goals were not changed on the system (8500 or 9000 steps in the goal setting group and no goals in the other 2 groups), and similarly, (2) the friend list was not changed (5 peers in the social comparison group, and no peers in the other 2 groups). The data collection was ended in May 2023.

### Ethical Considerations

The study protocol was approved by the Ethics Committee of the National Institute of Advanced Industrial Science and Technology (approval ID: 2022-1240). The protocol was preregistered with the Open Science Framework before data collection.

### Statistical Analyses

The primary outcome (ie, self-reported PA level) was analyzed using a 2-level multilevel model with the groups as between-person predictors, time (pre- vs postintervention time points) as the within-person predictor, and cross-level interactions. Groups were dummy coded, with the self-monitoring group as the reference level. Random intercepts and slopes were assumed, which allowed the baseline level of PA and rate of change in PA to vary across individuals. No correlation was assumed between the intercept and the slope. Each model was estimated using the restricted maximum likelihood estimation method. The same or similar models were used for the secondary outcomes. An exception is that the analyses of day-level variables (ie, daily step count and motivations reported each evening) had a continuous time predictor (ie, days since the start of the intervention) instead of pre- and postintervention time points. All randomized participants were included in the analyses on an intention-to-treat basis. The effect sizes (Cohen *d* for between-group differences and *dz* for within-group changes) were calculated [43]. All analyses were performed using R (version 4; R Foundation for Statistical Computing) with the specific packages *lme4* [44], *lmerTest* [45], and *fitbitr* [46].

Sensitivity analyses were planned to examine the influences of missingness on (1) the Fitbit-assessed step count and (2) evening levels of motivation for PA. First, for the main analysis on step count, we excluded days with steps <2000 as outliers. This cutoff was determined on the distribution observed in the current data (5.6% [110/1960] of records met <2000 steps). Also, 2000 steps can be reliably regarded as too few given that a daily step count of 5000 is operationalized as a sedentary lifestyle [47], and thus, measurement issues were suspected (eg, Fitbit was not worn appropriately). Second, as a sensitivity analysis, we reran the analysis while filtering out the days with missing heart rate (ie, days with <50% of valid heart rate records) instead of days with <2000 steps. Note that heart rate was not a target of our analyses but was measured by Fitbit; also, missingness in heart rate does not always indicate invalid wearing as temporary dysfunctions in the device or sensor may result in missingness. Third, we checked compliance in responding to daily questionnaires to see whether any participants should be excluded because of too few valid responses. However, we found excellent compliance in the current data (24.96/28 days, 89%, 1747/1960; see the *Results* section for details), and thus, data from all participants were used for statistical analyses.

## Results

### Demographics and Descriptive Statistics at the Baseline

The demographic characteristics of the participants are shown in Table 2. At the baseline assessment, the 70 participants were physically inactive: 46 (66%) in the precontemplation stage, 15 (21%) in the contemplation stage, and 9 (13%) in the preparation stage. In the IPAQ-SF, 46 (66%) participants did not meet the national health guidelines for PA (23 METs-h/w). Among the remaining participants (n=24), 10 (42%) were classified under category 3 (health-enhancing PA). As these highly active individuals were not the target of this study (and responded inconsistently to the SoC questionnaire and IPAQ-SF), we performed sensitivity analyses after excluding them at baseline (see Tables S4 and S5 in Multimedia Appendix 1 for details; overall, we identified no significant changes in the results that influenced the conclusion). No significant group differences were found in age, gender, education level, or other socioeconomic factors.

**Table 2:** Demographics and descriptives at baseline.

Variable		Self-monitoring (n=23)	Goal setting (n=23)	Social comparison (n=24)	Test on group differences, *F* test (*df*) or chi-square (*df*)		*P* value	
Age (years), mean (SD)		40.1 (11.4)	39.2 (12.8)	42.1 (13.7)	0.31 (2, 67)		.73	
Women, n (%)		15 (65.2)	17 (73.9)	12 (50.0)	2.96 (2)		.23	
BMI, mean (SD)		25.0 (4.5)	23.5 (5.5)	25.3 (4.1)	0.97 (2, 67)		.39	
Married, n (%)		13 (56.5)	11 (47.8)	17 (70.8)	2.62 (2)		.27	
Having a child, n (%)		13 (56.5)	13 (56.5)	14 (58.3)	0.02 (2)		.99	
**Education level, n (%)**						0.91 (4)		.92
	Middle or high school	10 (43.5)	11 (47.8)	13 (54.2)				
	College or vocational school	6 (26.1)	5 (21.7)	6 (25)				
	University and above	7 (30.4)	7 (30.4)	5 (20.8)				
Job, n (%)		17 (73.9)	19 (82.6)	20 (83.3)	0.78 (2)		.67	
**Household income, n (%)**						9.11 (8)		.33
	<3 million JPY (US $19,000)	7 (30.4)	3 (13.0)	6 (25)				
	3-5 million JPY (US $19,000-31,600)	8 (34.8)	13 (56.5)	7 (29.2)				
	5-7 million JPY (US $31,600-44,300)	5 (21.7)	4 (17.4)	7 (29.2)				
	7-10 million JPY (US $44,300-63,300)	2 (8.7)	0 (0)	3 (12.5)				
	>10 million JPY (US $63,300)	1 (4.4)	3 (13)	1 (4.2)				
Total PA^a^, mean (SD)		25.6 (33.7)	30.4 (46.9)	55.4 (128.1)	0.90 (2, 67)		.41	
**Stage of change, n (%)**						0.01 (4)		1.00
	Precontemplation	15 (65.2)	15 (65.2)	16 (66.7)				
	Contemplation	5 (21.7)	5 (21.7)	5 (20.8)				
	Preparation	3 (13)	3 (13)	3 (12.5)				
**SMSE-2^b^**								
	Intrinsic motivation	11.9 (3.2)	11.4 (3.8)	11.1 (3.7)	0.26 (2, 67)		.77	
	Integrated regulation	9.4 (3.6)	7.9 (2.7)	8.1 (3.6)	1.27 (2, 67)		.29	
	Identified regulation	13.7 (3.9)	13.8 (3.7)	13.0 (4.4)	0.27 (2, 67)		.76	
	Introjected regulation	10.4 (3.9)	8.5 (3.8)	8.0 (2.8)	2.97 (2, 67)		.06	
	External regulation	6.6 (2.2)	5.2 (2.3)	5.5 (3.0)	1.95 (2, 67)		.15	
	Amotivation	6.9 (2.7)	6.5 (3.4)	6.7 (2.7)	0.10 (2, 67)		.90	

^a^Total PA: total physical activity in metabolic equivalents for hours per week, as assessed using the International Physical Activity Questionnaire—Short Form.

^b^SMSE-2: Self-determined Motivation Scale for Exercise 2.

### Compliance and Adherence

**Figure 1:**
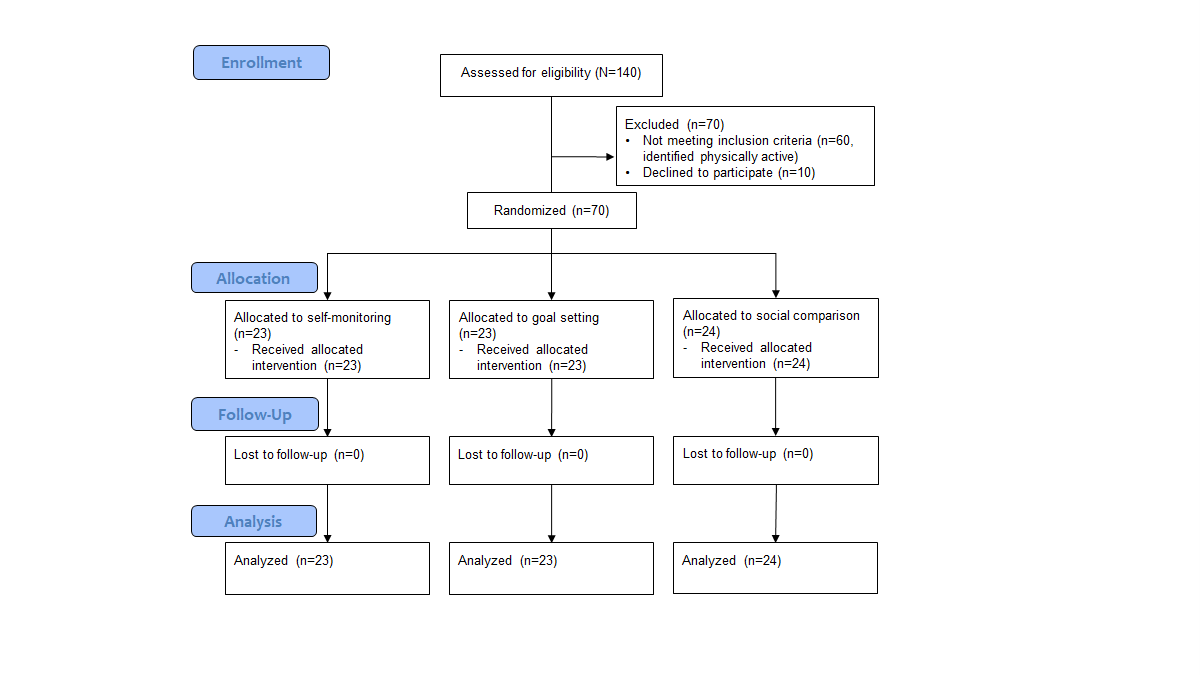
Consolidated Standards of Reporting Trials flow diagram.

All participants completed the pre- and postintervention assessments and received the intended interventions (see Figure 1 for the Consolidated Standards of Reporting Trials flow diagram). The compliance rate was 89% (mean 24.96, SD 5.75 out of 28 days) for the daily online questionnaires. The mean valid-wear time of Fitbit was 26.43 days (94%) out of the entire assessment period (28 days) after the invalid days (when <2000 steps were recorded) were left out. As another measure of adherence to wearing Fitbit, we checked the presence of heart rate signals in the data. This check identified 30 days to be excluded across 70 participants, and the remaining mean 27.57 (SD 1.04; 99%) days were submitted to the sensitivity analysis. No adverse event was recorded.

We examined the correlations between compliance and baseline variables (Table S1 in Multimedia Appendix 1). The older participants showed higher compliance (day-to-day assessments; *r*=0.30) and adherence (Fitbit wear time; *r*=0.39). Those with children or jobs showed higher adherence (*r*=0.35 and 0.31, respectively). Individuals with higher baseline levels of integrated regulations had a lower compliance rate (*r*=–0.35).

### Changes From the Pre- to Postintervention Assessments

The results of a 2 × 3 multilevel model (pre- vs postintervention time point; self-monitoring vs goal setting vs social comparison) of self-reported total PA revealed neither a significant main effect of time nor a significant interaction effect between time and group (Table 3). The same multilevel model analyses were repeated for SoC and motivation variables as outcomes, which showed no significant time-group interaction effects. However, significant effects of time were observed for the following secondary outcomes (Table S2 in Multimedia Appendix 1): SoC (*dz*=0.50, 95% CI 0.25-0.75, *P*=.006), integrated regulation (*dz*=0.35, 95% CI 0.11-0.59, *P*=.03), and external regulation (*dz*=0.42, 95% CI 0.17-0.66, *P*=.003). These time effects suggest that participants reported progress to higher stages at the end of the interventions; typically, people who had been in the precontemplation stage progressed toward the contemplation stage (see Table S3 in Multimedia Appendix 1). Similarly, participants experienced increases in integrated regulation (ie, they recognized more personal values and sense of self in PA) and external regulation (ie, they gained more motivation that was controlled by external sources and actions such as pleasing their family), but these effects did not differ between the BCT conditions.

**Table 3:** Changes in the levels of physical activity and other outcomes from the pre- to postintervention assessment.

Outcome and group^a^			Value, mean (SD)		Standardized within-group change (*dz*)		Standardized mean group difference (vs self-monitoring)		
							Cohen *d*		*P* value
**Total PA^b^ (METs-h/w)^c^**									
	Self-monitoring	–4.97 (27.82)		–0.18		—^d^		—	
	Goal	5.48 (46.49)		0.12		0.28		.33	
	Social	22.12 (80.80)		0.27		0.45		.15	
	All groups	7.75 (56.91)		0.14		—		—	
**SoC^e^**									
	Self-monitoring	0.48 (0.73)		0.65		—		—	
	Goal	0.52 (0.90)		0.58		0.05		.86	
	Social	0.21 (0.78)		0.27		–0.37		.25	
	All groups	0.40 (0.81)		0.50		—		—	
**Intrinsic motivation**									
	Self-monitoring	0.83 (3.26)		0.25		—		—	
	Goal	0.96 (2.42)		0.40		0.05		.88	
	Social	–0.58 (3.35)		0.17		–0.44		.12	
	All groups	0.39 (3.08)		0.13		—		—	
**Integrated regulation**									
	Self-monitoring	1.17 (2.85)		0.41		—		—	
	Goal	1.09 (2.29)		0.47		–0.03		.91	
	Social	0.38 (2.41)		0.16		–0.31		.28	
	All groups	0.87 (2.52)		0.35		—		—	
**Identified regulation**									
	Self-monitoring	1.39 (3.35)		0.41		—		—	
	Goal	0.96 (3.02)		0.32		–0.14		.68	
	Social	–0.13 (4.26)		0.03		–0.40		.15	
	All groups	0.73 (3.60)		0.20		—		—	
**Introjected regulation**									
	Self-monitoring	0.57 (3.87)		0.15		—		—	
	Goal	0.65 (3.75)		0.17		0.02		.93	
	Social	1.83 (3.06)		0.60		0.37		.23	
	All groups	1.03 (3.57)		0.29		—		—	
**External regulation**									
	Self-monitoring	1.65 (2.62)		0.63		—		—	
	Goal	1.48 (1.81)		0.82		–0.08		.82	
	Social	0.21 (3.15)		0.07		–0.51		.06	
	All groups	1.10 (2.64)		0.42		—		—	
**Amotivation**									
	Self-monitoring	–0.43 (2.59)		0.17		—		—	
	Goal	–0.57 (2.64)		0.21		–0.05		.87	
	Social	0.63 (2.60)		0.24		0.42		.17	
	All groups	–0.11 (2.63)		0.04		—		—	

^a^n=23, 23, and 24 for the self-monitoring, goal setting (goal), and social comparison (social) conditions, respectively. Stage of change coded as follows: precontemplation=1, contemplation=2, preparation=3. *P* values for group differences were calculated using multilevel modeling (ie, group-time interaction effects; see Table S2 in Multimedia Appendix 1 for details).

^b^PA: physical activity.

^c^METs-h/w: metabolic equivalents for hours per week

^d^Not available.

^e^SoC: Stage of Change.

### Day-Level Outcomes

**Figure 2:**
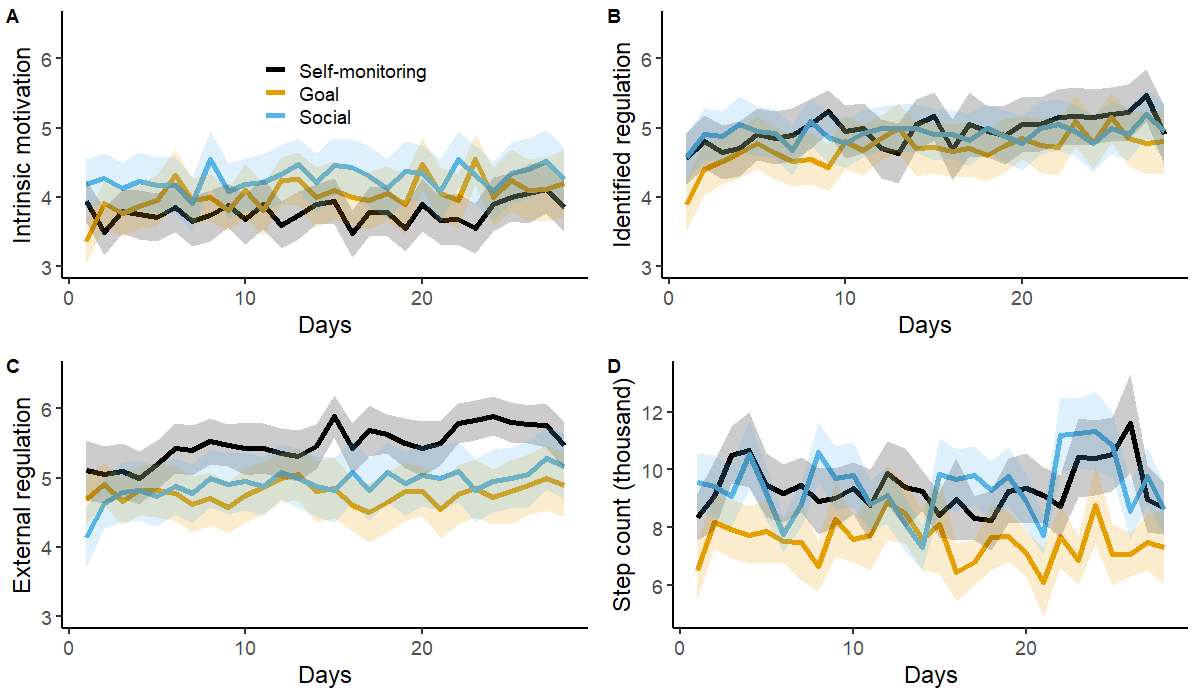
Levels of motivations and Fitbit-assessed step count as a function of day.

We performed multilevel modeling with days, groups, and their interactions as predictors. The results showed no significant day-group interactions in predicting Fitbit-assessed step count, identified regulation, or intrinsic motivation (Figure 2; Table 4). However, external motivation was significantly predicted by the interaction between the day and group dummy of self-monitoring versus goal setting. This interaction effect implies that the self-monitoring group experienced a significant increase in external motivation (*b*=0.019, SE=0.008; t_68.24_=2.436, *P*=.02), whereas the goal setting group showed no change over time (*b*=–0.003, SE=0.011; t_66.51_=–0.516, *P*=.61). Over the intervention period, the Fitbit-assessed step count remained slightly higher in the goal setting and social comparison conditions than in the self-monitoring condition (Figure 2D), although these main effects of the condition did not reach statistical significance (*P*=.051 and *P*=.06; Table 4). The results were unchanged when heart rate was used to define invalid wearing time (the main effects were not significant; *P*=.053 for goal setting and *P*=.17 for social comparison).

**Table 4:** Estimates of multilevel models predicting the day-level outcomes^a^.

IV^b^			Estimate		SE		2-tailed *t* test (*df*)		*P* value		95% CI
**DV^c^: identified regulation**											
	Time	0.011		0.008		1.307 (67.01)		.20		–0.005 to 0.027	
	Goal	–0.287		0.499		–0.575 (66.19)		.57		–1.257 to 0.684	
	Social	0.087		0.493		0.176 (66.13)		.86		–0.873 to 1.047	
	Time×Goal	0.001		0.012		0.047 (66.43)		.96		–0.022 to 0.023	
	Time×Social	–0.006		0.011		–0.500 (65.67)		.62		–0.028 to 0.017	
**DV: intrinsic motivation**											
	Time	0.011		0.008		1.403 (68.00)		.17		–0.004 to 0.027	
	Goal	0.175		0.503		0.348 (65.93)		.73		–0.803 to 1.153	
	Social	0.426		0.498		0.856 (65.87)		.40		–0.542 to 1.394	
	Time×Goal	–0.003		0.011		–0.236 (67.27)		.81		–0.025 to 0.019	
	Time×Social	–0.006		0.011		–0.516 (66.51)		.61		–0.027 to 0.016	
**DV: external regulation**											
	Time	0.019		0.008		2.436 (68.24)		.02		0.004 to 0.033	
	Goal	–0.385		0.519		–0.741 (66.25)		.46		–1.394 to 0.625	
	Social	–0.454		0.514		–0.883 (66.19)		.38		–1.453 to 0.545	
	Time×Goal	–0.022		0.011		–2.105 (67.47)		.04		–0.043 to –0.002	
	Time×Social	–0.002		0.010		–0.169 (66.70)		.87		–0.022 to 0.018	
**DV: Fitbit-assessed step count**											
	Time	–0.020		0.022		–0.923 (64.93)		.36		–0.063 to 0.022	
	Goal	0.238		0.120		1.990 (66.59)		.051		0.006 to 0.471	
	Social	0.221		0.114		1.933 (66.96)		.06		–0.002 to 0.444	
	Time×Goal	0.026		0.029		0.905 (63.13)		.37		–0.030 to 0.082	
	Time×Social	0.038		0.028		1.377 (64.72)		.17		–0.016 to 0.093	

^a^The step count was log transformed. Goal, Social=dummies representing the goal setting or social comparison conditions vs self-monitoring. A total of 1934 and 1960 observations were used for the motivation outcomes and step count, respectively.

^b^IV: independent variable.

^c^DV: dependent variable.

## Discussion

### Principal Findings

This study examined the effects of different BCTs implemented in Fitbit on subjective and objective levels of PA as well as motivations for PA. Three BCTs (ie, self-monitoring, goal setting, and social comparison) were offered via the smartwatch for 4 weeks, during which participants lived in their daily lives. Overall, participants showed excellent adherence to the interventions (older participants and those with a child or job showed even better adherence), and there were no dropouts at the end of the interventions. Although we found no significant increase in total PA (as assessed by the IPAQ-SF) or Fitbit-assessed step count, participants reported that the stage progressed typically from precontemplation to contemplation across the 3 BCT conditions. In addition, significant increases were detected in several aspects of motivation (ie, integrated and external regulation), and the day-to-day level of external regulation showed a larger increase in self-monitoring than in goal-setting conditions.

Overall, these results suggest that the effects of Fitbit-delivered BCTs on PA are marginal. There are several possible reasons for the detection of smaller-than-expected effects. First, we used an active control condition (ie, self-monitoring offered by a wearable device), unlike previous trials that compared the presence and absence of an activity tracker [10], which may have mitigated the detectable between-condition effects. Second, changes from the pre- to the postintervention assessment showed extremely large heterogeneity. This heterogeneity can be partly attributed to large individual differences (or SD) in baseline PA levels. Notably, the social comparison group showed an average increase of 22.1 (SD 80.9) METs-h/w, which is regarded as a sufficient increase in PA according to the national health recommendation (23 METs-h/w) [32]. However, a large SD prevented the effect from reaching statistical significance, which is in line with a recent meta-analysis of the heterogeneous effects of social factors in behavior change interventions [26]. This may indicate that personalization (delivering social comparisons to people who appreciate social factors) is important for maximizing the intervention effect [48]. Third, we did not have a baseline period for the Fitbit assessment; on receipt of the Fitbit, the participants started each intervention immediately. Interestingly, the step count was slightly higher in the goal setting and social comparison groups than in the self-monitoring group over the course of the intervention (*P*=.051 and *P*=.06). These effects suggest that participants had already adapted their behavior on the first day of the interventions, which resulted in an overall elevation in step count, albeit with nonsignificant time-group interactions. These minor effects should be interpreted carefully, but it is possible that self-regulatory and social factors contribute a meaning addition to self-monitoring in PA promotion [24].

Our aim was to experimentally investigate the effects of mobile implementations of BCTs, and therefore, we decided to keep each BCT manipulation as simple as possible. However, our implementations might feel too simple compared with the features and functions equipped in commercial PA apps. For example, we set rigid goals for each participant (ie, 8000 or 9500 steps each day), but goal setting theory suggests that goals should be adequate and tailored to the individual skills (ie, doable but challenging) [25,49]. Also, other components could be considered as effective additions in real-life implementation, such as action planning, goal evaluation, and goal reevaluation. Similarly, social comparison could be provided in another form—for example, some participants commented in the postintervention interview that they would be more motivated if the leaderboard displayed the steps of close others (eg, real friends and family members). Several trials have tested the efficacy of collaboration and competition within a gamification intervention for PA, which gives a meaningful context of social comparison and may help enhance motivation to engage in the intervention [50,51].

We detected significant increases in the levels of integrated and external regulations in the postintervention assessment. These increases were not moderated by the BCT conditions, indicating that each group experienced almost equal increases in the 2 types of regulation. Similarly, day-level external regulation increased over the 4-week interventions, which was qualified by the significant time-group interaction. This interaction suggests that participants in the self-monitoring group experienced a larger increase in external regulation than those in the goal setting group. A cross-sectional survey of the Mexican general population [52] found that external regulation was predominant in the precontemplation stage, whereas intrinsic, integrated, and identified regulations increased as the stage progressed (the action and maintenance stage). The detected increase in integrated regulation may be a good sign, but the increase in external regulation may have both positive (eg, people may have found external rewards to start PA and to progress from the precontemplation stage) and negative aspects (eg, this may not directly lead to habit formation). Furthermore, the day-to-day increase in external regulation may suggest that participants in the self-monitoring group felt some pressure and tension regarding PA, as the questionnaire item stated that they felt that they have to exercise [30]. Thus, the significant interaction effect can be interpreted as the goal setting technique preventing participants from feeling obliged to engage in PA.

### Limitations

These findings should be interpreted with consideration of several important limitations. First, we based the eligibility assessment on a 1-item SoC questionnaire, which is not an optimal screening tool. Approximately one-third of eligible participants were sufficiently active to exceed the national health recommendation (23 METs-h/w) at baseline. In the postintervention interview, some participants reported that they had not been aware of their activity level at eligibility screening but realized how active they were when responding to each item of the IPAQ-SF. Future research should consider a sufficiently detailed eligibility assessment for PA and preferably use an objective measure, such as a pedometer or accelerometer. Second, it was not possible to strictly control how the participants used each Fitbit feature during the intervention period. For example, we did not prevent participants from setting their own step goals, even in the self-monitoring or social comparison conditions. Although we did not detect any significant violations in the log of Fitbit usage, it might be appropriate to add a system to lock or unlock a particular function in the app and device to reduce potential contamination. Third, we did not conduct a follow-up assessment to test whether the intervention effects were maintained even after the end of the intervention. This decision was made because we could not give away the Fitbit, but it would be valid to leave the Fitbit with participants even after the end of the intervention if we wanted to observe a long-lasting follow-up effect. Fourth, during the study period, motivation was rated or reported each night together with the step count of the day. This assessment timing may have influenced how participants responded to the questionnaires—it is possible that people are more motivated for PA during the daytime. However, we decided not to administer multiple questionnaires (eg, in the morning; at random moments) within a day to reduce the burden on participants and to achieve high compliance.

### Conclusions

Nevertheless, our results suggest that activity trackers are useful for increasing readiness and motivation for PA, although significant heterogeneity was identified. The addition of goal setting and social comparison potentially enhances step count, and goal setting may prevent feelings of pressure or tension from engaging in PA. However, to fully detect BCT-specific effects, a proper trial (eg, with a larger sample size, longer intervention and assessment periods, and different populations) is warranted. It is difficult to evaluate the effects of each BCT and their complex combinations (eg, [53]), but future research should expand to other BCTs often used in mHealth practices, such as messaging and rewards.

## Appendix

Multimedia Appendix 1 Supplementary tables.

Multimedia Appendix 2 CONSORT checklist.

## Abbreviations

BCT: behavior change technique
IPAQ-SF: International Physical Activity Questionnaire—Short Form
METs-h/w: hourly metabolic equivalents per week
PA: physical activity
SMD: standardized mean difference
SMSE: Self-determined Motivation Scale for Exercise
SoC: Stage of Change
