# Supplementary material for: Deconstructing Fitbit to Specify the Effective Features in Promoting Physical Activity Among Inactive Adults: Pilot Randomized Controlled Trial
Source: JMIR Mhealth Uhealth. 2024 Jul 12;12:e51216. doi: 10.2196/51216 (PMC11282379; doi:10.2196/51216)
Supplement: Multimedia Appendix 1 [file mhealth_v12i1e51216_app1.docx]

**Multimedia Appendix 1**

**Table S1.** Correlations between Baseline Variables and Compliance (Day-to-day Assessments) and Adherence (Fitbit Wear Time)

| Baseline variable | Compliance rate | Adherence rate |
| --- | --- | --- |
| Age | 0.30 | 0.39 |
| Sex | 0.07 | 0.16 |
| BMI | 0.09 | -0.19 |
| Marital status | 0.20 | 0.13 |
| Child | 0.10 | 0.35 |
| Education level | -0.13 | 0.03 |
| Job | 0.04 | 0.31 |
| Household income | 0.28 | 0.05 |
| PA | 0.08 | 0.11 |
| SoC | -0.06 | 0.08 |
| Intrinsic motivation | -0.07 | -0.06 |
| Integrated regulation | -0.35 | 0.07 |
| Identified regulation | -0.01 | -0.01 |
| Introjected regulation | -0.21 | 0.00 |
| External regulation | 0.15 | 0.11 |
| Amotivation | 0.08 | 0.14 |
| Compliance rate | 1.00 | 0.07 |
| Adherence rate | 0.07 | 1.00 |

*Note.* Sex: coded as men = 0, women = 1; PA = Physical activity; SoC = Stage of change, coded as follows: Precontemplation = 1; Contemplation = 2; Preparation = 3. Collapsed across conditions.

**Table S2.** Multilevel Models on the Outcomes Assessed at the Pre- and Post-intervention Assessments

| IV | Estimate | *SE* | *t* | *p* | CI  2.5% | CI 97.5% |
| --- | --- | --- | --- | --- | --- | --- |
| DV: Total PA |  |  |  |  |  |  |
| Time | 0.10 | 0.32 | 0.31 | 0.76 | -0.52 | 0.72 |
| Goal | -0.05 | 0.48 | -0.10 | 0.92 | -0.98 | 0.88 |
| Social | 0.13 | 0.48 | 0.27 | 0.78 | -0.79 | 1.05 |
| Time × Goal | 0.44 | 0.45 | 0.97 | 0.33 | -0.44 | 1.32 |
| Time × Social | 0.65 | 0.45 | 1.46 | 0.15 | -0.22 | 1.52 |
| DV: SoC |  |  |  |  |  |  |
| Time | 0.48 | 0.17 | 2.85 | 0.01 | 0.15 | 0.80 |
| Goal | 0.00 | 0.20 | 0.00 | 1.00 | -0.39 | 0.39 |
| Social | -0.02 | 0.20 | -0.10 | 0.92 | -0.40 | 0.36 |
| Time × Goal | 0.04 | 0.24 | 0.18 | 0.86 | -0.42 | 0.51 |
| Time × Social | -0.27 | 0.23 | -1.15 | 0.25 | -0.73 | 0.19 |
| DV: Intrinsic motivation | | |  |  |  |  |
| Time | 0.83 | 0.63 | 1.30 | 0.20 | -0.41 | 2.06 |
| Goal | -0.48 | 1.05 | -0.45 | 0.65 | -2.53 | 1.57 |
| Social | -0.74 | 1.04 | -0.71 | 0.48 | -2.77 | 1.28 |
| Time × Goal | 0.13 | 0.90 | 0.15 | 0.88 | -1.61 | 1.87 |
| Time × Social | -1.41 | 0.89 | -1.59 | 0.12 | -3.14 | 0.32 |
| DV: Integrated regulation | | |  |  |  |  |
| Time | 1.17 | 0.53 | 2.23 | 0.03 | 0.15 | 2.20 |
| Goal | -1.43 | 0.97 | -1.47 | 0.15 | -3.32 | 0.45 |
| Social | -1.22 | 0.96 | -1.27 | 0.21 | -3.09 | 0.65 |
| Time × Goal | -0.09 | 0.75 | -0.12 | 0.91 | -1.54 | 1.36 |
| Time × Social | -0.80 | 0.74 | -1.08 | 0.28 | -2.23 | 0.64 |
| DV: Identified regulation | | |  |  |  |  |
| Time | 1.39 | 0.75 | 1.86 | 0.07 | -0.07 | 2.85 |
| Goal | 0.09 | 1.18 | 0.07 | 0.94 | -2.20 | 2.37 |
| Social | -0.70 | 1.16 | -0.60 | 0.55 | -2.96 | 1.57 |
| Time × Goal | -0.43 | 1.06 | -0.41 | 0.68 | -2.49 | 1.63 |
| Time × Social | -1.52 | 1.05 | -1.45 | 0.15 | -3.55 | 0.52 |
| DV: Introjected regulation | | |  |  |  |  |
| Time | 0.57 | 0.74 | 0.76 | 0.45 | -0.88 | 2.01 |
| Goal | -1.87 | 1.04 | -1.80 | 0.08 | -3.89 | 0.15 |
| Social | -2.39 | 1.03 | -2.33 | 0.02 | -4.39 | -0.39 |
| Time × Goal | 0.09 | 1.05 | 0.08 | 0.93 | -1.96 | 2.13 |
| Time × Social | 1.27 | 1.04 | 1.22 | 0.23 | -0.76 | 3.29 |
| DV: External regulation | | |  |  |  |  |
| Time | 1.65 | 0.54 | 3.05 | <0.01 | 0.60 | 2.70 |
| Goal | -1.43 | 0.75 | -1.90 | 0.06 | -2.90 | 0.03 |
| Social | -1.07 | 0.75 | -1.43 | 0.16 | -2.52 | 0.38 |
| Time × Goal | -0.17 | 0.76 | -0.23 | 0.82 | -1.66 | 1.31 |
| Time × Social | -1.44 | 0.76 | -1.91 | 0.06 | -2.92 | 0.03 |
| DV: Amotivation | |  |  |  |  |  |
| Time | -0.43 | 0.54 | -0.80 | 0.43 | -1.49 | 0.62 |
| Goal | -0.39 | 0.87 | -0.45 | 0.65 | -2.08 | 1.29 |
| Social | -0.20 | 0.86 | -0.24 | 0.81 | -1.87 | 1.46 |
| Time × Goal | -0.13 | 0.77 | -0.17 | 0.87 | -1.63 | 1.37 |
| Time × Social | 1.06 | 0.76 | 1.39 | 0.17 | -0.42 | 2.54 |

*Note.* N = 23, 23, and 24 for the self-monitoring, goal-setting (Goal) and social-comparison (Social) condition. SoC = Stage of change, coded as follows: Precontemplation = 1; Contemplation = 2; Preparation = 3.

**Table S3.** Number of Participants Identified at each Stage of Change

| Condition | Time | Precontemplation | Contemplation | Preparation |
| --- | --- | --- | --- | --- |
| Self-monitoring | Baseline | 15 | 5 | 3 |
|  | Post-intervention | 4 | 16 | 3 |
| Goal setting | Baseline | 15 | 5 | 3 |
|  | Post-intervention | 4 | 15 | 4 |
| Social comparison | Baseline | 16 | 5 | 3 |
|  | Post-intervention | 11 | 10 | 3 |

*Note.* No participants were identified to be at the action or higher stages.

**Table S4.** Multilevel Models on the Outcomes Assessed at the Pre- and Post-intervention Timing (Active Participants at the Baseline being Excluded)

| IV | Estimate | *SE* | *t* | *p* | CI  2.5% | CI 97.5% |
| --- | --- | --- | --- | --- | --- | --- |
| DV: Total PA |  |  |  |  |  |  |
| Time | 0.22 | 0.33 | 0.65 | 0.52 | -0.43 | 0.87 |
| Goal | -0.21 | 0.46 | -0.46 | 0.65 | -1.09 | 0.68 |
| Social | -0.29 | 0.46 | -0.63 | 0.53 | -1.19 | 0.60 |
| Time × Goal | 0.53 | 0.48 | 1.11 | 0.27 | -0.40 | 1.46 |
| Time × Social | 0.80 | 0.49 | 1.66 | 0.10 | -0.14 | 1.75 |
| DV: SoC |  |  |  |  |  |  |
| Time | 0.57 | 0.17 | 3.40 | <0.01 | 0.24 | 0.90 |
| Goal | 0.07 | 0.21 | 0.33 | 0.74 | -0.34 | 0.47 |
| Social | 0.20 | 0.21 | 0.93 | 0.35 | -0.21 | 0.61 |
| Time × Goal | -0.02 | 0.24 | -0.09 | 0.93 | -0.49 | 0.45 |
| Time × Social | -0.47 | 0.24 | -1.91 | 0.06 | -0.94 | 0.01 |
| DV: Intrinsic motivation | | |  |  |  |  |
| Time | 1.19 | 0.68 | 1.76 | 0.08 | -0.12 | 2.50 |
| Goal | -0.57 | 1.15 | -0.50 | 0.62 | -2.80 | 1.66 |
| Social | -0.25 | 1.16 | -0.22 | 0.83 | -2.51 | 2.01 |
| Time × Goal | -0.24 | 0.97 | -0.25 | 0.80 | -2.12 | 1.64 |
| Time × Social | -1.93 | 0.98 | -1.96 | 0.05 | -3.83 | -0.02 |
| DV: Integrated regulation | | |  |  |  |  |
| Time | 1.19 | 0.57 | 2.10 | 0.04 | 0.09 | 2.29 |
| Goal | -1.74 | 1.06 | -1.63 | 0.11 | -3.80 | 0.32 |
| Social | -0.97 | 1.08 | -0.90 | 0.37 | -3.06 | 1.12 |
| Time × Goal | 0.01 | 0.81 | 0.01 | 0.99 | -1.57 | 1.58 |
| Time × Social | -0.87 | 0.82 | -1.06 | 0.29 | -2.47 | 0.72 |
| DV: Identified regulation | | |  |  |  |  |
| Time | 1.52 | 0.82 | 1.86 | 0.07 | -0.07 | 3.11 |
| Goal | 0.42 | 1.29 | 0.33 | 0.74 | -2.08 | 2.92 |
| Social | -0.17 | 1.31 | -0.13 | 0.90 | -2.70 | 2.37 |
| Time × Goal | -0.72 | 1.17 | -0.62 | 0.54 | -3.00 | 1.55 |
| Time × Social | -1.21 | 1.19 | -1.02 | 0.31 | -3.51 | 1.10 |
| DV: Introjected regulation | | |  |  |  |  |
| Time | 0.57 | 0.75 | 0.76 | 0.45 | -0.88 | 2.03 |
| Goal | -2.14 | 1.12 | -1.90 | 0.06 | -4.31 | 0.03 |
| Social | -2.19 | 1.14 | -1.92 | 0.06 | -4.39 | 0.01 |
| Time × Goal | 0.33 | 1.07 | 0.31 | 0.76 | -1.76 | 2.41 |
| Time × Social | 1.85 | 1.09 | 1.70 | 0.09 | -0.26 | 3.96 |
| DV: External regulation | | |  |  |  |  |
| Time | 1.76 | 0.54 | 3.24 | <0.01 | 0.71 | 2.82 |
| Goal | -1.67 | 0.79 | -2.11 | 0.04 | -3.20 | -0.13 |
| Social | -1.19 | 0.80 | -1.49 | 0.14 | -2.75 | 0.36 |
| Time × Goal | -0.36 | 0.78 | -0.47 | 0.64 | -1.87 | 1.15 |
| Time × Social | -1.39 | 0.79 | -1.77 | 0.08 | -2.92 | 0.14 |
| DV: Amotivation | |  |  |  |  |  |
| Time | -0.38 | 0.51 | -0.74 | 0.46 | -1.37 | 0.61 |
| Goal | -0.95 | 0.87 | -1.10 | 0.28 | -2.64 | 0.73 |
| Social | -0.54 | 0.88 | -0.61 | 0.54 | -2.24 | 1.17 |
| Time × Goal | 0.38 | 0.73 | 0.52 | 0.60 | -1.04 | 1.80 |
| Time × Social | 1.07 | 0.74 | 1.44 | 0.16 | -0.38 | 2.51 |

*Note.* N = 21, 20, and 19 for the self-monitoring, goal-setting (Goal) and social-comparison (Social) condition. SoC = Stage of change, coded as follows: Precontemplation = 1; Contemplation = 2; Preparation = 3.

**Table S5.** Estimates of Multilevel Models Predicting the Day-level Outcomes (Active Participants at the Baseline being Excluded)

| IV | Estimate | *SE* | *t* | *p* | 95%CI |
| --- | --- | --- | --- | --- | --- |
| DV: Identified regulation |  |  |  |  |  |
| Time | 0.010 | 0.009 | 1.057 | 0.295 | [-0.008,0.027] |
| Goal | -0.222 | 0.562 | -0.395 | 0.695 | [-1.313,0.870] |
| Social | 0.229 | 0.570 | 0.403 | 0.689 | [-0.876,1.335] |
| Time × Goal | -0.003 | 0.013 | -0.220 | 0.827 | [-0.028,0.022] |
| Time × Social | -0.006 | 0.013 | -0.439 | 0.663 | [-0.031,0.019] |
| DV: Intrinsic motivation |  |  |  |  |  |
| Time | 0.011 | 0.008 | 1.304 | 0.197 | [-0.005,0.027] |
| Goal | 0.198 | 0.572 | 0.346 | 0.730 | [-0.912,1.308] |
| Social | 0.394 | 0.579 | 0.680 | 0.499 | [-0.731,1.518] |
| Time × Goal | -0.007 | 0.012 | -0.602 | 0.549 | [-0.030,0.016] |
| Time × Social | -0.004 | 0.012 | -0.348 | 0.729 | [-0.027,0.019] |
| DV: External regulation |  |  |  |  |  |
| Time | 0.018 | 0.008 | 2.244 | 0.029 | [0.002,0.034] |
| Goal | -0.355 | 0.575 | -0.617 | 0.540 | [-1.472,0.763] |
| Social | -0.450 | 0.583 | -0.773 | 0.443 | [-1.582,0.682] |
| Time × Goal | -0.022 | 0.011 | -1.942 | 0.057 | [-0.044,0.000] |
| Time × Social | 0.001 | 0.012 | 0.089 | 0.930 | [-0.021,0.023] |
| DV: Fitbit-assessed step count |  |  |  |  |  |
| Time | -0.020 | 0.023 | -0.880 | 0.383 | [-0.066,0.025] |
| Goal | 0.163 | 0.121 | 1.348 | 0.183 | [-0.072,0.398] |
| Social | 0.109 | 0.115 | 0.947 | 0.348 | [-0.114,0.333] |
| Time × Goal | 0.014 | 0.032 | 0.428 | 0.670 | [-0.048,0.075] |
| Time × Social | 0.046 | 0.030 | 1.502 | 0.139 | [-0.013,0.105] |

*Note.* N = 21, 20, and 19 for the self-monitoring, goal-setting (Goal) and social-comparison (Social) condition.
